# Supplementary material for: Temporal progression along discrete coding states during decision-making in the mouse gustatory cortex
Source: PLoS Comput Biol. 2023 Feb 7;19(2):e1010865. doi: 10.1371/journal.pcbi.1010865 (PMC9904478; doi:10.1371/journal.pcbi.1010865)
Supplement: S1 Fig — Given a state S, the probability of observing S in a correctly performed trial was compared across the four trial types using a Chi-squared test. If the result was not significant (N), S was labeled “Non-coding.” Otherwise (Y), all six pairwise Marascuilo post-hoc tests were performed. If there was exactly one trial type for which all post-hoc tests involving it were significant (Y), S was labeled “Taste ID-coding.” Otherwise (N), the four trial types were grouped in two orthogonal ways: sweet (sucrose and maltose) and bitter (quinine and sucrose octaacetate), and cue left (sucrose and quinine) and cue right (maltose and sucrose octaacetate). Two additional Chi-squared tests were run: one to compare the probability of observing S in correctly performed trials between the sweet and bitter categories, and another to compare the probability of observing S in correctly performed trials between the cue left and cue right categories. These two tests are run independently, leading to four possible significance outcomes. If neither test was significant, S was labeled “Non-coding.” If both tests were significant, S was labeled “Dual-coding.” If the sweet vs. bitter test was significant and the cue left vs. cue right test was not, S was labeled “Quality-coding.” If the sweet vs. bitter test was not significant and the cue left vs. cue right test was, S was labeled “Decision-coding,” but could be further classified by examining its frequency of occurrence in correctly vs. incorrectly performed trials. If the cued direction for which S was more likely to be observed in correctly performed trials was the same for incorrectly performed trials (referred to as the “preferred direction” for S in correct and incorrect trials), S was labeled “Cue-coding.” If the preferred directions in correct and incorrect trials were opposite, S was labeled “Action-coding”. (PDF) [file pcbi.1010865.s001.pdf]

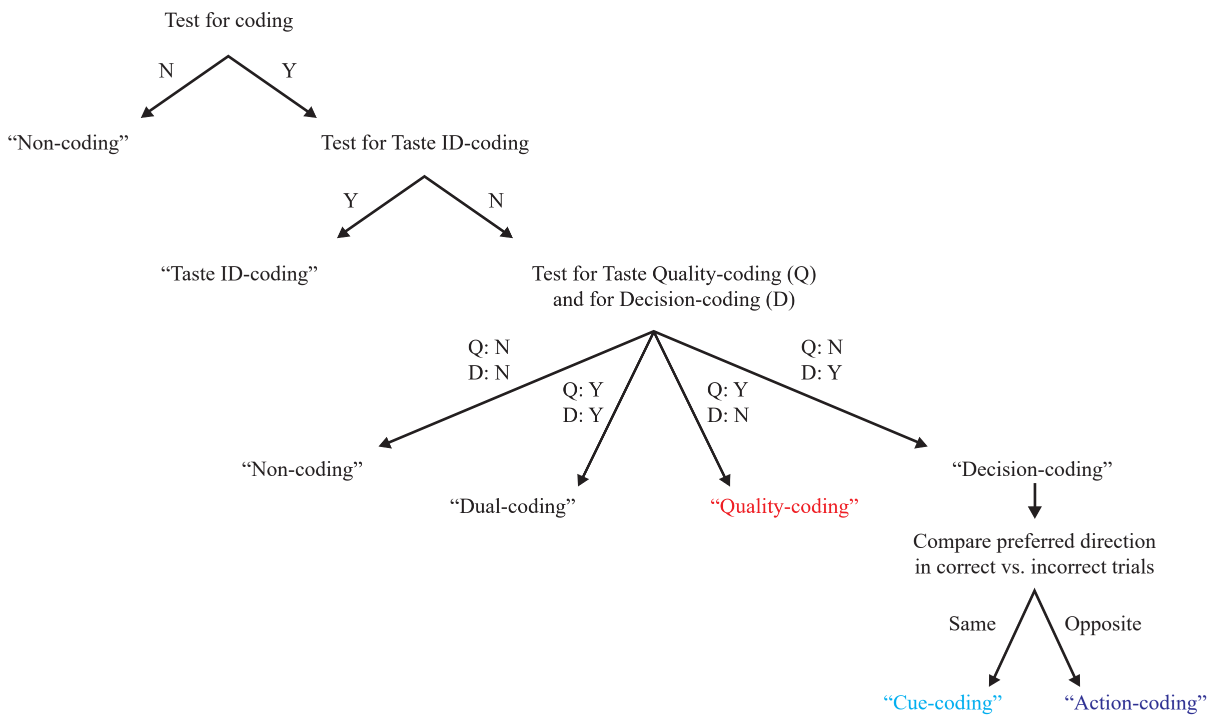

S1 Fig. Pipeline for classification of HMM states. Given a state S, the probability of observing S in a correctly performed trial was compared across the four trial types using a Chi-squared test. If the result was not significant (N), S was labeled "Non-coding." Otherwise (Y), all six pairwise Marascuilo post-hoc tests were performed. If there was exactly one trial type for which all post-hoc tests involving it were significant (Y), S was labeled "Taste ID-coding." Otherwise (N), the four trial types were grouped in two orthogonal ways: sweet (sucrose and maltose) and bitter (quinine and sucrose octaacetate), and cue left (sucrose and quinine) and cue right (maltose and sucrose octaacetate). Two additional Chi-squared tests were run: one to compare the probability of observing S in correctly performed trials between the sweet and bitter categories, and another to compare the probability of observing S in correctly performed trials between the cue left and cue right categories. These two tests are run independently, leading to four possible significance outcomes. If neither test was significant, S was labeled "Non-coding." If both tests were significant, S was labeled "Dual-coding." If the sweet vs. bitter test was significant and the cue left vs. cue right test was not, S was labeled "Quality-coding." If the sweet vs. bitter test was not significant and the cue left vs. cue right test was, S was labeled "Decision-coding," but could be further classified by examining its frequency of occurrence in correctly vs. incorrectly performed trials. If the cued direction for which S was more likely to be observed in correctly performed trials was the same for incorrectly performed trials (referred to as the "preferred direction" for S in correct and incorrect trials), S was labeled "Cue-coding." If the preferred directions in correct and incorrect trials were opposite, S was labeled "Action-coding."
