# Supplementary material for: Temporal progression along discrete coding states during decision-making in the mouse gustatory cortex
Source: PLoS Comput Biol. 2023 Feb 7;19(2):e1010865. doi: 10.1371/journal.pcbi.1010865 (PMC9904478; doi:10.1371/journal.pcbi.1010865)
Supplement: S2 Fig — A: Generic synaptic structure between clusters without task roles. Top: Cartoon representing a pair of E-I partner clusters with their synaptic connection weights (red, flathead arrows: inhibitory connections; black, pointed arrows: excitatory connections). Bottom: synaptic matrix for the generic pair of E-I partner clusters (colormap corresponding to units of pA×ms). Given any two excitatory clusters, E1 and E2, and their inhibitory partners, I1 and I2, synaptic connections were strong between neurons within the same cluster or belonging to partner clusters (JE++JEE or JI++Jαβ, with α, β ∈ {E, I}), and weak between neurons in different clusters (JE−JEE or JI−Jαβ, with α, β ∈ {E, I}). B: Top: Venn diagram depicting the “overlapping” structure of two E-I taste cluster pairs with the same quality (sweet or bitter). Bottom: synaptic matrix of “overlapping” taste clusters depicted at top (colormap corresponding to units of pA×ms). Taste neurons in different clusters with the same taste quality had strongly connected overlapping subclusters (E1∩E2 and I1∩I2 in the Venn diagram), with synaptic strength JE++JEE or JI++Jαβ, with α, β ∈ {E, I}. These overlapping subclusters are shown in the bottom right corner of each taste cluster in the synaptic matrix. Taste neurons in the same cluster but outside the overlapping subclusters were connected with synaptic strength JE+JEE or JI+Jαβ, with α, β ∈ {E, I}. All other neurons were connected as in the generic clusters of panel A, with JE− < 1 < JE+ < JE++ and JI− < 1 < JI+ < JI++. (PDF) [file pcbi.1010865.s002.pdf]

Synaptic structure for taste clusters

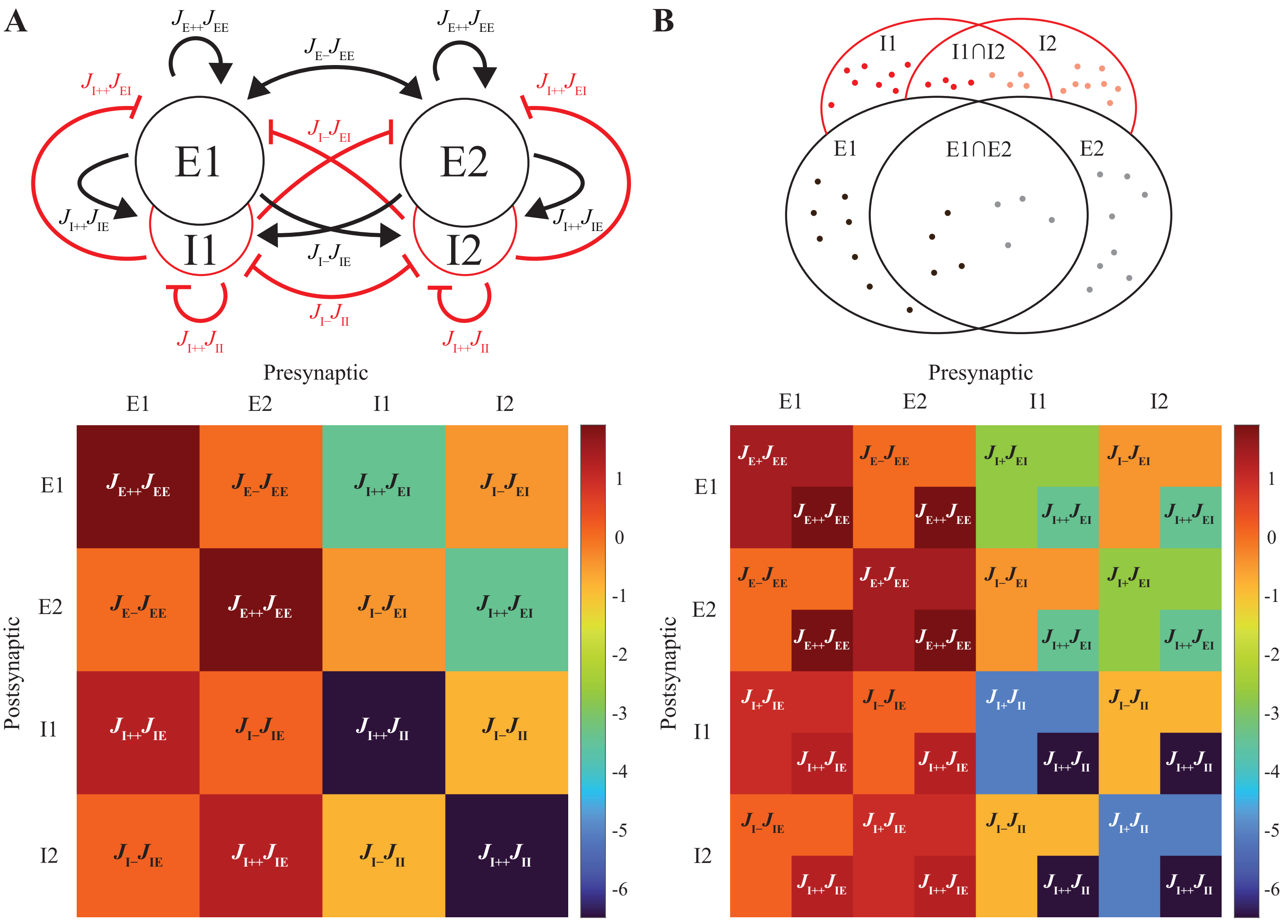

S2 Fig. Synaptic structure for taste clusters. **A**: Generic synaptic structure between clusters without task roles. **Top**: Cartoon representing a pair of E-I partner clusters with their synaptic connection weights (red, flathead arrows: inhibitory connections; black, pointed arrows: excitatory connections). **Bottom**: synaptic matrix for the generic pair of E-I partner clusters (colormap corresponding to units of pA×ms). Given any two excitatory clusters, E1 and E2, and their inhibitory partners, I1 and I2, synaptic connections were strong between neurons within the same cluster or belonging to partner clusters ($J_{E++}J_{EE}$ or $J_{I++}J_{\alpha\beta}$, with $\alpha, \beta \in \{E, I\}$), and weak between neurons in different clusters ($J_{E-}J_{EE}$ or $J_{I-}J_{\alpha\beta}$, with $\alpha, \beta \in \{E, I\}$). **B**: **Top**: Venn diagram depicting the "overlapping" structure of two E-I taste cluster pairs with the same quality (sweet or bitter). **Bottom**: synaptic matrix of "overlapping" taste clusters depicted at top (colormap corresponding to units of pA×ms). Taste neurons in different clusters with the same taste quality had strongly connected overlapping subclusters (E1∩E2 and I1∩I2 in the Venn diagram), with synaptic strength $J_{E++}J_{EE}$ or $J_{I++}J_{\alpha\beta}$, with $\alpha, \beta \in \{E, I\}$. These overlapping subclusters are shown in the bottom right corner of each taste cluster in the synaptic matrix. Taste neurons in the same cluster but outside the overlapping subclusters were connected with synaptic strength $J_{E+}J_{EE}$ or $J_{I+}J_{\alpha\beta}$, with $\alpha, \beta \in \{E, I\}$. All other neurons were connected as in the generic clusters of panel **A**, with $J_{E-} < 1 < J_{E+} < J_{E++}$ and $J_{I-} < 1 < J_{I+} < J_{I++}$.
