# Supplementary material for: Temporal progression along discrete coding states during decision-making in the mouse gustatory cortex
Source: PLoS Comput Biol. 2023 Feb 7;19(2):e1010865. doi: 10.1371/journal.pcbi.1010865 (PMC9904478; doi:10.1371/journal.pcbi.1010865)
Supplement: S3 Fig — The main synaptic connections are depicted by curved lines (pointed arrowheads are excitatory connections, flat arrowheads are inhibitory connections). The strength of each connection is indicated next to the corresponding curved line (see S3 Table for parameter values). Synaptic weights from excitatory taste clusters to appropriate excitatory cue clusters (i.e., S → CL, M → CR, Q → CL, and O → CR) were larger by a factor JCT,E compared to generic weights between different clusters (e.g., S → CR, M → CL, Q → CR, and O → CL). Excitatory cue clusters had stronger intracluster connections (by a factor JCC,E) compared to generic clusters; the synaptic weights from an inhibitory cue cluster to the opposite excitatory cue cluster were larger by a factor JCC,I. Connections from excitatory cue clusters to correct excitatory action clusters (i.e., CL → AL and CR → AR) were larger by a factor JAC,Cor, while the connections to incorrect excitatory action clusters (i.e., CL → AR and CR → AL) were larger by a factor JAC,Inc, with JAC,Cor > JAC,Inc. Excitatory action clusters had stronger intracluster connections (scaled up by JAA,E) and the weights from their inhibitory partners to the opposite excitatory action cluster were scaled up by JAA,I. Connections from inhibitory action clusters to excitatory cue clusters were magnified by JCA,I. All synaptic weight modifiers were applied with a given probability (reported in S3 Table). Connections among clusters not explicitly depicted here were as in S2 Fig (also see S4 Fig for the full synaptic weight matrix). Key: S: sucrose, M: maltose, Q: quinine, O: octaacetate, CL: cue left, CR: cue right, AL: action left, AR: action right. (PDF) [file pcbi.1010865.s003.pdf]

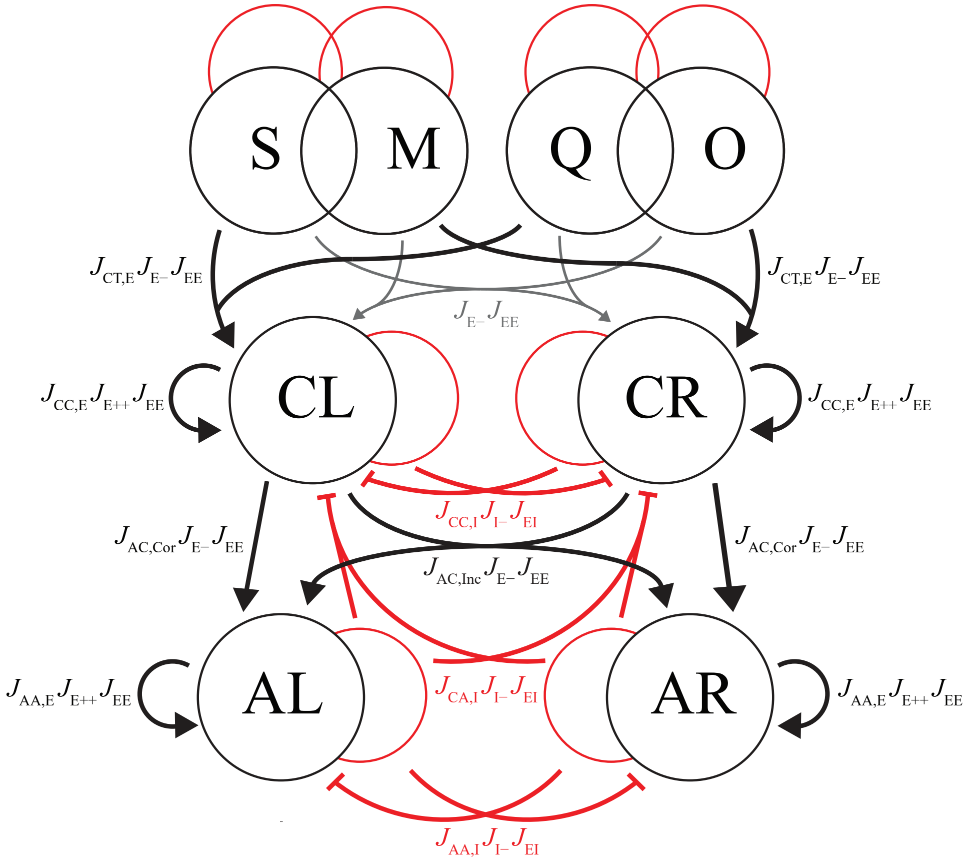

S3 Fig. Details of synaptic connections among taste, cue, and action E-I cluster pairs (compare with main **Fig 3B**). The main synaptic connections are depicted by curved lines (pointed arrowheads are excitatory connections, flat arrowheads are inhibitory connections). The strength of each connection is indicated next to the corresponding curved line (see **S3 Table** for parameter values). Synaptic weights from excitatory taste clusters to appropriate excitatory cue clusters (i.e., S $\to$ CL, M $\to$ CR, Q $\to$ CL, and O $\to$ CR) were larger by a factor $J_{\mathrm{CT,E}}$ compared to generic weights between different clusters (e.g., S $\to$ CR, M $\to$ CL, Q $\to$ CR, and O $\to$ CL). Excitatory cue clusters had stronger intracluster connections (by a factor $J_{\mathrm{CC,E}}$) compared to generic clusters; the synaptic weights from an inhibitory cue cluster to the opposite excitatory cue cluster were larger by a factor $J_{\mathrm{CC,I}}$. Connections from excitatory cue clusters to correct excitatory action clusters (i.e., CL $\to$ AL and CR $\to$ AR) were larger by a factor $J_{\mathrm{AC,Cor}}$, while the connections to incorrect excitatory action clusters (i.e., CL $\to$ AR and CR $\to$ AL) were larger by a factor $J_{\mathrm{AC,Inc}}$, with $J_{\mathrm{AC,Cor}} > J_{\mathrm{AC,Inc}}$. Excitatory action clusters had stronger intracluster connections (scaled up by $J_{\mathrm{AA,E}}$) and the weights from their inhibitory partners to the opposite excitatory action cluster were scaled up by $J_{\mathrm{AA,I}}$. Connections from inhibitory action clusters to excitatory cue clusters were magnified by $J_{\mathrm{CA,I}}$. All synaptic weight modifiers were applied with a given probability (reported in **S3 Table**). Connections among clusters not explicitly depicted here were as in **S2 Fig** (also see **S4 Fig** for the full synaptic weight matrix). Key: S: sucrose, M: maltose, Q: quinine, O: octaacetate, CL: cue left, CR: cue right, AL: action left, AR: action right.
