# Supplementary material for: Temporal progression along discrete coding states during decision-making in the mouse gustatory cortex
Source: PLoS Comput Biol. 2023 Feb 7;19(2):e1010865. doi: 10.1371/journal.pcbi.1010865 (PMC9904478; doi:10.1371/journal.pcbi.1010865)
Supplement: S4 Fig — The value in a block occupying row i and column j is the average synaptic weight (in units of pA×ms) from a presynaptic neuron belonging to cluster j to a postsynaptic neuron belonging to cluster i. The average synaptic values shown in the table were obtained (up to rounding) as: Pαβ[fov2Jγ+++(1-fov2)JγX]Jαβ for connections involving taste clusters with the same taste quality (sweet or bitter), and Pαβ(PMJM + 1 − PM)JγY Jαβ for all other connections. Here α, β, γ ∈ {E, I}, γ = E if and only if α = β = E, X ∈ {−, +}, Y ∈ {−, ++ }, JM ∈ {JCT,E, JCC,E, JCC,I, JAC,Cor, JAC,Inc, JAA,E, JAA,I, JCA,I} is a weight modifier, and PM ∈ {0, PCT,E, PCC,E, PCC,I, PAC,Cor, PAC,Inc, PAA,E, PAA,I, PCA,I} is the corresponding probability of applying the modifier (PM = 0 when modifier was not applied). Key: S: sucrose cluster, M: maltose cluster, Q: quinine cluster, O: octaacetate cluster, CL: cue left cluster, CR: cue right cluster, AL: action left cluster, AR: action right cluster, 9–14: clusters without task roles, B: background (excitatory) population. (PDF) [file pcbi.1010865.s004.pdf]

Full synaptic matrix

Presynaptic (columns); Postsynaptic (rows)

	S	Q	M	O	CL	CR	AL	AR	9	10	11	12	13	14	B	S	Q	M	O	CL	CR	AL	AR	9	10	11	12	13	14
S	0.296	0.009	0.033	0.009	0.009	0.009	0.009	0.009	0.009	0.009	0.009	0.009	0.009	0.009	0.009	-1.370	-0.211	-0.307	-0.211	-0.211	-0.211	-0.211	-0.211	-0.211	-0.211	-0.211	-0.211	-0.211	-0.211
Q	0.009	0.296	0.009	0.033	0.009	0.009	0.009	0.009	0.009	0.009	0.009	0.009	0.009	0.009	0.009	-0.211	-1.370	-0.211	-0.307	-0.211	-0.211	-0.211	-0.211	-0.211	-0.211	-0.211	-0.211	-0.211	-0.211
M	0.033	0.009	0.296	0.009	0.009	0.009	0.009	0.009	0.009	0.009	0.009	0.009	0.009	0.009	0.009	-0.307	-0.211	-1.370	-0.211	-0.211	-0.211	-0.211	-0.211	-0.211	-0.211	-0.211	-0.211	-0.211	-0.211
O	0.009	0.033	0.009	0.296	0.009	0.009	0.009	0.009	0.009	0.009	0.009	0.009	0.009	0.009	0.009	-0.211	-0.307	-0.211	-1.370	-0.211	-0.211	-0.211	-0.211	-0.211	-0.211	-0.211	-0.211	-0.211	-0.211
CL	0.013	0.013	0.009	0.009	0.396	0.009	0.009	0.009	0.009	0.009	0.009	0.009	0.009	0.009	0.009	-0.211	-0.211	-0.211	-0.211	-1.725	-0.254	-0.253	-0.254	-0.211	-0.211	-0.211	-0.211	-0.211	-0.211
CR	0.009	0.009	0.013	0.013	0.009	0.396	0.009	0.009	0.009	0.009	0.009	0.009	0.009	0.009	0.009	-0.211	-0.211	-0.211	-0.211	-0.254	-1.725	-0.254	-0.254	-0.211	-0.211	-0.211	-0.211	-0.211	-0.211
AL	0.009	0.009	0.009	0.009	0.016	0.015	0.397	0.009	0.009	0.009	0.009	0.009	0.009	0.009	0.009	-0.211	-0.211	-0.211	-0.211	-0.211	-0.211	-1.725	-0.423	-0.211	-0.211	-0.211	-0.211	-0.211	-0.211
AR	0.009	0.009	0.009	0.009	0.015	0.016	0.009	0.397	0.009	0.009	0.009	0.009	0.009	0.009	0.009	-0.211	-0.211	-0.211	-0.211	-0.211	-0.211	-0.423	-1.725	-0.211	-0.211	-0.211	-0.211	-0.211	-0.211
9	0.009	0.009	0.009	0.009	0.009	0.009	0.009	0.009	0.384	0.009	0.009	0.009	0.009	0.009	0.009	-0.211	-0.211	-0.211	-0.211	-0.211	-0.211	-0.211	-0.211	-1.725	-0.211	-0.211	-0.211	-0.211	-0.211
10	0.009	0.009	0.009	0.009	0.009	0.009	0.009	0.009	0.009	0.384	0.009	0.009	0.009	0.009	0.009	-0.211	-0.211	-0.211	-0.211	-0.211	-0.211	-0.211	-0.211	-0.211	-1.725	-0.211	-0.211	-0.211	-0.211
11	0.009	0.009	0.009	0.009	0.009	0.009	0.009	0.009	0.009	0.009	0.384	0.009	0.009	0.009	0.009	-0.211	-0.211	-0.211	-0.211	-0.211	-0.211	-0.211	-0.211	-0.211	-0.211	-1.725	-0.211	-0.211	-0.211
12	0.009	0.009	0.009	0.009	0.009	0.009	0.009	0.009	0.009	0.009	0.009	0.384	0.009	0.009	0.009	-0.211	-0.211	-0.211	-0.211	-0.211	-0.211	-0.211	-0.211	-0.211	-0.211	-0.211	-1.725	-0.211	-0.211
13	0.009	0.009	0.009	0.009	0.009	0.009	0.009	0.009	0.009	0.009	0.009	0.009	0.384	0.009	0.009	-0.211	-0.211	-0.211	-0.211	-0.211	-0.211	-0.211	-0.211	-0.211	-0.211	-0.211	-0.211	-1.725	-0.211
14	0.009	0.009	0.009	0.009	0.009	0.009	0.009	0.009	0.009	0.009	0.009	0.009	0.009	0.384	0.009	-0.211	-0.211	-0.211	-0.211	-0.211	-0.211	-0.211	-0.211	-0.211	-0.211	-0.211	-0.211	-0.211	-1.725
B	0.009	0.009	0.009	0.009	0.009	0.009	0.009	0.009	0.009	0.009	0.009	0.009	0.009	0.009	0.009	-0.211	-0.211	-0.211	-0.211	-0.211	-0.211	-0.211	-0.211	-0.211	-0.211	-0.211	-0.211	-0.211	-0.211
S	0.494	0.076	0.111	0.076	0.076	0.076	0.076	0.076	0.076	0.076	0.076	0.076	0.076	0.076	0.076	-2.611	-0.403	-0.585	-0.403	-0.403	-0.403	-0.403	-0.403	-0.403	-0.403	-0.403	-0.403	-0.403	-0.403
Q	0.076	0.494	0.076	0.111	0.076	0.076	0.076	0.076	0.076	0.076	0.076	0.076	0.076	0.076	0.076	-0.403	-2.611	-0.403	-0.585	-0.403	-0.403	-0.403	-0.403	-0.403	-0.403	-0.403	-0.403	-0.403	-0.403
M	0.111	0.076	0.494	0.076	0.076	0.076	0.076	0.076	0.076	0.076	0.076	0.076	0.076	0.076	0.076	-0.585	-0.403	-2.611	-0.403	-0.403	-0.403	-0.403	-0.403	-0.403	-0.403	-0.403	-0.403	-0.403	-0.403
O	0.076	0.111	0.076	0.494	0.076	0.076	0.076	0.076	0.076	0.076	0.076	0.076	0.076	0.076	0.076	-0.403	-0.585	-0.403	-2.611	-0.403	-0.403	-0.403	-0.403	-0.403	-0.403	-0.403	-0.403	-0.403	-0.403
CL	0.076	0.076	0.076	0.076	0.621	0.076	0.076	0.076	0.076	0.076	0.076	0.076	0.076	0.076	0.076	-0.403	-0.403	-0.403	-0.403	-3.286	-0.403	-0.403	-0.403	-0.403	-0.403	-0.403	-0.403	-0.403	-0.403
CR	0.076	0.076	0.076	0.076	0.076	0.621	0.076	0.076	0.076	0.076	0.076	0.076	0.076	0.076	0.076	-0.403	-0.403	-0.403	-0.403	-0.403	-3.286	-0.403	-0.403	-0.403	-0.403	-0.403	-0.403	-0.403	-0.403
AL	0.076	0.076	0.076	0.076	0.076	0.076	0.621	0.076	0.076	0.076	0.076	0.076	0.076	0.076	0.076	-0.403	-0.403	-0.403	-0.403	-0.403	-0.403	-3.286	-0.403	-0.403	-0.403	-0.403	-0.403	-0.403	-0.403
AR	0.076	0.076	0.076	0.076	0.076	0.076	0.076	0.621	0.076	0.076	0.076	0.076	0.076	0.076	0.076	-0.403	-0.403	-0.403	-0.403	-0.403	-0.403	-0.403	-3.286	-0.403	-0.403	-0.403	-0.403	-0.403	-0.403
9	0.076	0.076	0.076	0.076	0.076	0.076	0.076	0.076	0.621	0.076	0.076	0.076	0.076	0.076	0.076	-0.403	-0.403	-0.403	-0.403	-0.403	-0.403	-0.403	-0.403	-3.286	-0.403	-0.403	-0.403	-0.403	-0.403
10	0.076	0.076	0.076	0.076	0.076	0.076	0.076	0.076	0.076	0.621	0.076	0.076	0.076	0.076	0.076	-0.403	-0.403	-0.403	-0.403	-0.403	-0.403	-0.403	-0.403	-0.403	-3.286	-0.403	-0.403	-0.403	-0.403
11	0.076	0.076	0.076	0.076	0.076	0.076	0.076	0.076	0.076	0.076	0.621	0.076	0.076	0.076	0.076	-0.403	-0.403	-0.403	-0.403	-0.403	-0.403	-0.403	-0.403	-0.403	-0.403	-3.286	-0.403	-0.403	-0.403
12	0.076	0.076	0.076	0.076	0.076	0.076	0.076	0.076	0.076	0.076	0.076	0.621	0.076	0.076	0.076	-0.403	-0.403	-0.403	-0.403	-0.403	-0.403	-0.403	-0.403	-0.403	-0.403	-0.403	-3.286	-0.403	-0.403
13	0.076	0.076	0.076	0.076	0.076	0.076	0.076	0.076	0.076	0.076	0.076	0.076	0.621	0.076	0.076	-0.403	-0.403	-0.403	-0.403	-0.403	-0.403	-0.403	-0.403	-0.403	-0.403	-0.403	-0.403	-3.286	-0.403
14	0.076	0.076	0.076	0.076	0.076	0.076	0.076	0.076	0.076	0.076	0.076	0.076	0.076	0.621	0.076	-0.403	-0.403	-0.403	-0.403	-0.403	-0.403	-0.403	-0.403	-0.403	-0.403	-0.403	-0.403	-0.403	-3.286

S4 Fig. Full synaptic weight matrix of the network model. The value in a block occupying row i and column j is the *average* synaptic weight (in units of pA×ms) from a presynaptic neuron belonging to cluster j to a postsynaptic neuron belonging to cluster i. The average synaptic values shown in the table were obtained (up to rounding) as: $P_{\alpha\beta}[f_{\rm ov}^2 J_{\gamma++} + (1-f_{\rm ov}^2)J_{\gamma X}]J_{\alpha\beta}$ for connections involving taste clusters with the same taste quality (sweet or bitter), and $P_{\alpha\beta}(P_M J_M + 1 - P_M)J_{\gamma Y}J_{\alpha\beta}$ for all other connections. Here $\alpha, \beta, \gamma \in \{\mathrm{E}, \mathrm{I}\}$, $\gamma = \mathrm{E}$ if and only if $\alpha = \beta = \mathrm{E}$, $X \in \{-, +\}$, $Y \in \{-, ++\}$, $J_M \in \{J_{\rm CT,E},\ J_{\rm CC,E},\ J_{\rm CC,I},\ J_{\rm AC,Cor},\ J_{\rm AC,Inc},\ J_{\rm AA,E},\ J_{\rm AA,I},\ J_{\rm CA,I}\}$ is a weight modifier, and $P_M \in \{0,\ P_{\rm CT,E},\ P_{\rm CC,E},\ P_{\rm CC,I},\ P_{\rm AC,Cor},\ P_{\rm AC,Inc},\ P_{\rm AA,E},\ P_{\rm AA,I},\ P_{\rm CA,I}\}$ is the corresponding probability of applying the modifier ($P_M = 0$ when modifier was not applied). Key: S: sucrose cluster, M: maltose cluster, Q: quinine cluster, O: octaacetate cluster, CL: cue left cluster, CR: cue right cluster, AL: action left cluster, AR: action right cluster, 9-14: clusters without task roles, B: background (excitatory) population.
