# Supplementary material for: Temporal progression along discrete coding states during decision-making in the mouse gustatory cortex
Source: PLoS Comput Biol. 2023 Feb 7;19(2):e1010865. doi: 10.1371/journal.pcbi.1010865 (PMC9904478; doi:10.1371/journal.pcbi.1010865)
Supplement: S5 Fig — A, C: Effect of simulated silencing during sampling and delay periods on task performance for models with stimulus input with gain 60% and decay time constant 160 ms (A) and 705 ms (C), respectively. B, D: Distribution of onset times of coding states after fitting HMMs to models with stimulus input as in corresponding left panel. * indicates significant difference (p < 0.05) for Bonferroni-corrected post-hoc test vs. None condition after significant within-subjects ANOVA across the 3 conditions. N.S. indicates no significant difference. (PDF) [file pcbi.1010865.s005.pdf]

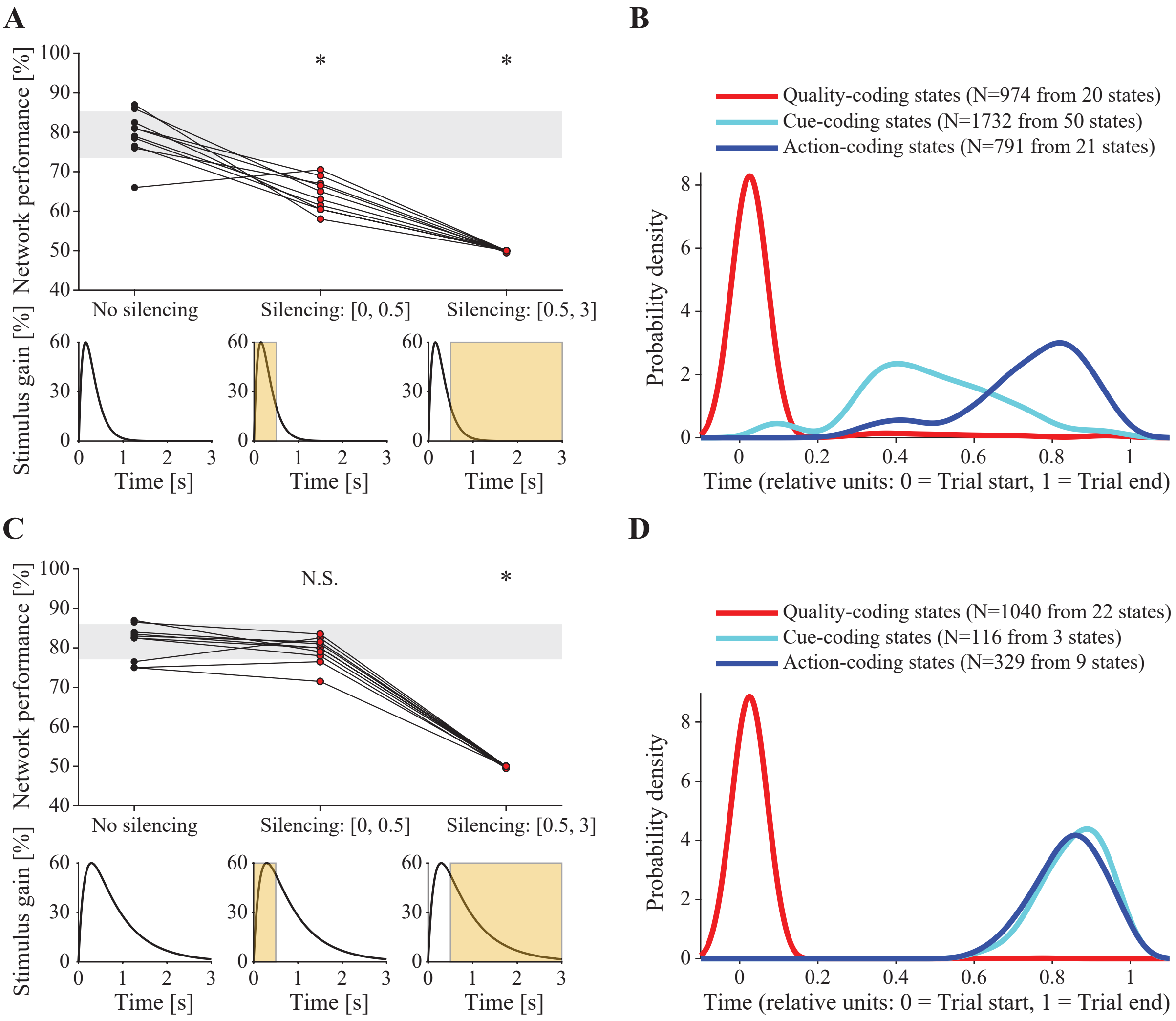

S5 Fig. Network's performance and onset times of coding states for different parameters of the stimulus input (supplements main **Fig 6**). **A**, **C**: Effect of simulated silencing during sampling and delay periods on task performance for models with stimulus input with gain 60% and decay time constant 160 ms (**A**) and 705 ms (**C**), respectively. **B**, **D**: Distribution of onset times of coding states after fitting HMMs to models with stimulus input as in corresponding left panel. * indicates significant difference ($p < 0.05$) for Bonferroni-corrected post-hoc test vs. None condition after significant within-subjects ANOVA across the 3 conditions. N.S. indicates no significant difference.
