# Supplementary material for: Temporal progression along discrete coding states during decision-making in the mouse gustatory cortex
Source: PLoS Comput Biol. 2023 Feb 7;19(2):e1010865. doi: 10.1371/journal.pcbi.1010865 (PMC9904478; doi:10.1371/journal.pcbi.1010865)
Supplement: S6 Fig — Silencing was implemented as a square pulse stimulus with width 250 ms and height (strength, 100% or 25%) determining the increase in baseline external current for all inhibitory neurons in the network. Each point is an average accuracy over 1, 000 trials (100 trials from each of 10 networks) for silencing centered at that point. For all points, the input stimulus had a gain of 200% and decay time constant of 160 ms. Grey region represents the mean ±1 standard deviation of accuracy for networks with no silencing (same as in main Fig 6B). Regions-of-interest for silencing employed in main Fig 7A (Beginning, Cue onset, and Middle) are shaded in yellow. (PDF) [file pcbi.1010865.s006.pdf]

Model performance as a function of silencing time period

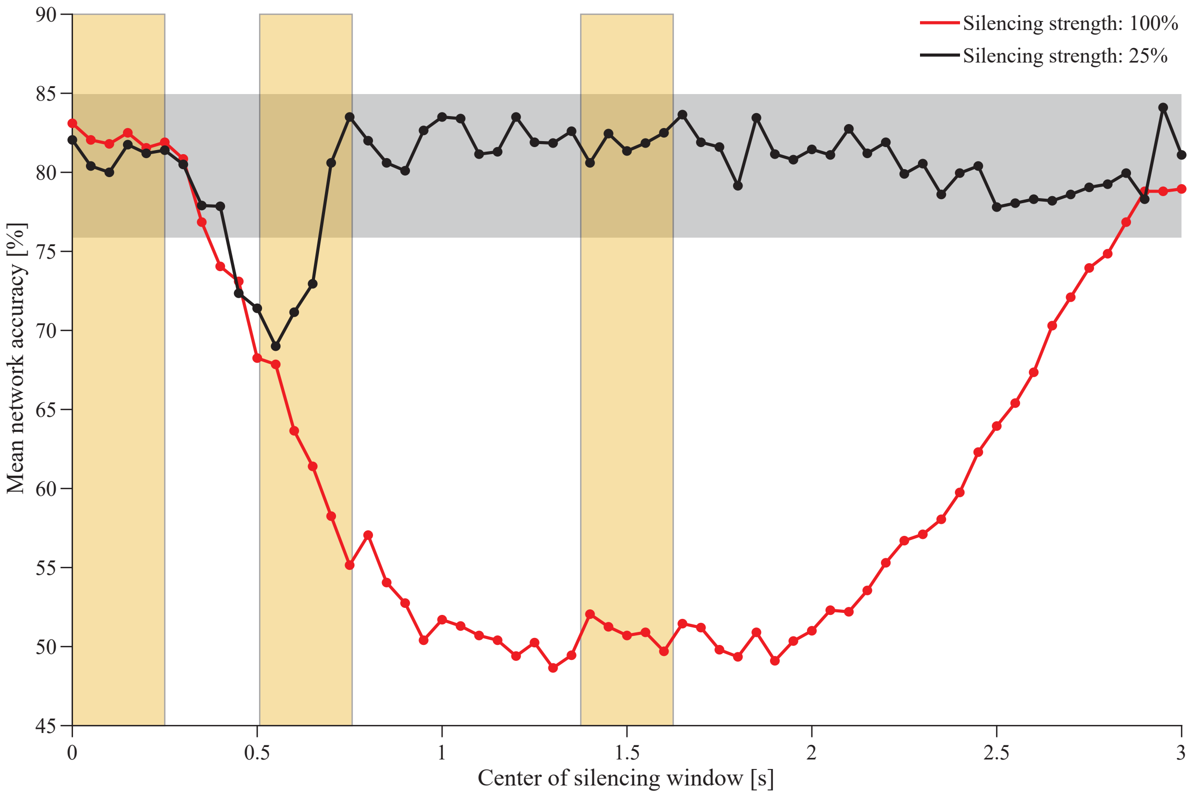

S6 Fig. Effect of simulated optogenetic silencing on network task performance as a function of time of silencing. Silencing was implemented as a square pulse stimulus with width 250 ms and height (strength, 100% or 25%) determining the increase in baseline external current for all inhibitory neurons in the network. Each point is an average accuracy over 1,000 trials (100 trials from each of 10 networks) for silencing centered at that point. For all points, the input stimulus had a gain of 200% and decay time constant of 160 ms. Grey region represents the mean ±1 standard deviation of accuracy for networks with no silencing (same as in main **Fig 6B**). Regions-of-interest for silencing employed in main **Fig 7A** (Beginning, Cue onset, and Middle) are shaded in yellow.
