# Supplementary material for: Temporal progression along discrete coding states during decision-making in the mouse gustatory cortex
Source: PLoS Comput Biol. 2023 Feb 7;19(2):e1010865. doi: 10.1371/journal.pcbi.1010865 (PMC9904478; doi:10.1371/journal.pcbi.1010865)
Supplement: S1 Table — Also indicated is the distribution of these states over sessions (16 sessions total). The same coding state classification procedure (see S1 Fig) was applied to the trial-by-trial decoding results from each model. “Hidden states” is the theoretical best number determined from model fitting; “Decoded states” is how many were actually found after decoding trial-by-trial. (PDF) [file pcbi.1010865.s007.pdf]

Hidden states in data

EXPERIMENT	Model fit to experimental data	Model fit to circularly shuffled data	Model fit to swap-shuffled data
Hidden states	Mean: 5.6<br>Median: 5<br>Range: 3 – 9	Mean: 5.1<br>Median: 5<br>Range: 2 – 8	Mean: 3.6<br>Median: 3<br>Range: 2 – 7
Decoded states	Total: 77<br>Mean: 4.8<br>Median: 4.5<br>Range: 3 – 8	Total: 62<br>Mean: 3.9<br>Median: 4<br>Range: 2 – 7	Total: 41<br>Mean: 2.6<br>Median: 2<br>Range: 1 – 4
Decision-coding states	15 (over 10 sessions)	14 (over 8 sessions)	10 (over 6 sessions)
Cue-coding states	8 (over 7 sessions)	9 (over 5 sessions)	3 (over 3 sessions)
Action-coding states	7 (over 5 sessions)	5 (over 5 sessions)	7 (over 5 sessions)
Quality-coding states	4 (over 4 sessions)	5 (over 5 sessions)	4 (over 3 sessions)
Taste ID-coding states	8 (over 6 sessions)	10 (over 6 sessions)	9 (over 6 sessions)
Dual-coding states	3 (over 2 sessions)	2 (over 1 session)	3 (over 2 sessions)
Non-coding states	47 (over 15 sessions)	31 (over 12 sessions)	15 (over 9 sessions)
Sessions with Quality- and Decision-coding states	3	1	1
Sessions with Cue- and Action-coding states	2	2	2
Sessions with Quality-, Cue-, and Action-coding states	1	1	0

S1 Table. Summary of the numbers of states found by HMM models fit to unshuffled, circularly shuffled, and swap-shuffled experimental data. Also indicated is the distribution of these states over sessions (16 sessions total). The same coding state classification procedure (see **S1 Fig**) was applied to the trial-by-trial decoding results from each model. “Hidden states” is the theoretical best number determined from model fitting; “Decoded states” is how many were actually found after decoding trial-by-trial.
