# Supplementary material for: Temporal progression along discrete coding states during decision-making in the mouse gustatory cortex
Source: PLoS Comput Biol. 2023 Feb 7;19(2):e1010865. doi: 10.1371/journal.pcbi.1010865 (PMC9904478; doi:10.1371/journal.pcbi.1010865)
Supplement: S2 Table — Also indicated is the distribution of these states over sessions (10 sessions total). The same coding state classification procedure (see S1 Fig) was applied to the trial-by-trial decoding results from each model. “Hidden states” is the theoretical best number determined from model fitting; “Decoded states” is how many were actually found after decoding trial-by-trial. (PDF) [file pcbi.1010865.s008.pdf]

Hidden states in model

SIMULATION	Model fit to simulation data	Model fit to circularly shuffled data	Model fit to swap-shuffled data
Hidden states	Mean: 21.2<br>Median: 21<br>Range: 17 – 25	Mean: 18<br>Median: 17<br>Range: 14 – 24	Mean: 10.6<br>Median: 11<br>Range: 8 – 14
Decoded states	Total: 174<br>Mean: 17.4<br>Median: 17<br>Range: 15 – 21	Total: 158<br>Mean: 15.8<br>Median: 15.5<br>Range: 13 – 20	Total: 81<br>Mean: 8.1<br>Median: 8<br>Range: 6 – 10
Decision-coding states	83 (over 10 sessions)	74 (over 10 sessions)	4 (over 3 sessions)
Cue-coding states	50 (over 10 sessions)	52 (over 10 sessions)	1
Action-coding states	21 (over 10 sessions)	15 (over 8 sessions)	1
Quality-coding states	20 (over 10 sessions)	2 (over 2 sessions)	0
Taste ID-coding states	39 (over 10 sessions)	49 (over 10 sessions)	46 (over 10 sessions)
Dual-coding states	0	0	1
Non-coding states	32 (over 10 sessions)	33 (over 9 sessions)	30 (over 10 sessions)
Sessions with Quality- and Decision-coding states	10	2	0
Sessions with Cue- and Action-coding states	10	8	0
Sessions with Quality-, Cue-, and Action-coding states	10	1	0

S2 Table. Summary of numbers of states found by HMM models fit to unshuffled, circularly shuffled, and swap-shuffled simulation data. Also indicated is the distribution of these states over sessions (10 sessions total). The same coding state classification procedure (see **S1 Fig**) was applied to the trial-by-trial decoding results from each model. "Hidden states" is the theoretical best number determined from model fitting; "Decoded states" is how many were actually found after decoding trial-by-trial.
