# Supplementary material for: Temporal progression along discrete coding states during decision-making in the mouse gustatory cortex
Source: PLoS Comput Biol. 2023 Feb 7;19(2):e1010865. doi: 10.1371/journal.pcbi.1010865 (PMC9904478; doi:10.1371/journal.pcbi.1010865)
Supplement: S3 Table — Symbols, values, and brief descriptions for all parameters used in the simulations of the spiking network model presented in the main text. (PDF) [file pcbi.1010865.s009.pdf]

Model Parameters

**Symbol**	**Value**	**Name/Description**
NEURONS AND NETWORK MODEL		
V_{L}	0 mV	Resting membrane potential
V_{th}	20 mV	Spiking threshold
V_{r}	0 mV	Reset membrane potential
C	1 pF	Membrane capacitance
$\tau_{\mathrm{m,E}}$	20 ms	E membrane time constant
$\tau_{\mathrm{m,I}}$	10 ms	I membrane time constant
$\tau_{\mathrm{syn,E}}$	3 ms	E synaptic time constant
$\tau_{\mathrm{syn,CueE}}$	6.9 ms	E synaptic time constant for neurons in cue clusters
$\tau_{\mathrm{syn,ActE}}$	9.9 ms	E synaptic time constant for neurons in action clusters
$\tau_{\mathrm{syn,I}}$	2 ms	I synaptic time constant
$\tau_{\mathrm{syn,CueI}}$	2.6 ms	I synaptic time constant for neurons in cue clusters
$\tau_{\mathrm{syn,ActI}}$	6.5 ms	I synaptic time constant for neurons in action clusters
τ_{r}	5 ms	Absolute refractory period
N_{e}	4,000	Number of E neurons
N_{i}	994	Number of I neurons
$I^{*}_{\mathrm{E,ext}}$	2.05 pA	Baseline external input current for E neurons
$I^{*}_{\mathrm{I,ext}}$	2.16 pA	Baseline external input current for I neurons
Q	14	Number of clusters
f_{bg}	0.125	Fraction of E neurons in "background" population
f_{ov}	0.25	Fraction of neurons in E and I taste clusters assigned to "overlapping" subpopulations
t_{Total}	3 s	Trial simulation length
dt	0.05 ms	Simulation time step
P_{ee}	0.2	Probability of E $\to$ E connection
P_{ei}	0.5	Probability of I $\to$ E connection
P_{ie}	0.5	Probability of E $\to$ I connection
P_{ii}	0.5	Probability of I $\to$ I connection
J_{EE}	0.16 pA$\times$ms	E$\to$E synaptic weight (reference value)
J_{EI}	-0.63 pA$\times$ms	I$\to$E synaptic weight (reference value)
J_{IE}	0.23 pA$\times$ms	E$\to$I synaptic weight (reference value)
J_{II}	1.2 pA$\times$ms	I$\to$I synaptic weight (reference value)
$J_{\mathrm{E++}}$	12	E$\to$E maximum intracluster potentiation factor
$J_{\mathrm{E+}}$	$0.25 \times J_{\mathrm{E-}} + 0.75 \times J_{\mathrm{E++}}$	E$\to$E intermediate intracluster potentiation factor
$J_{\mathrm{E-}}$	0.267	E$\to$E generic depression factor for synaptic weights between neurons in different clusters
$J_{\mathrm{I++}}$	5.4	I$\to$E, E$\to$I, I$\to$I maximum intracluster potentiation factor
$J_{\mathrm{I+}}$	$0.25 \times J_{\mathrm{I-}} + 0.75 \times J_{\mathrm{E++}}$	I$\to$E, E$\to$I, I$\to$I intermediate potentiation factor
$J_{\mathrm{I-}}$	0.685	I$\to$E, E$\to$I, I$\to$I generic depression factor for synaptic weights between neurons in different clusters
$J_{\mathrm{CT,E}}$; $P_{\mathrm{CT,E}}$	1.85 ; 0.60	Weight modifier for connections from E taste cluster to appropriate E cue cluster; Probability of applying modifier
$J_{\mathrm{CC,E}}$; $P_{\mathrm{CC,E}}$	1.04 ; 0.60	Weight modifier for connections from E cue cluster to itself; Probability of applying modifier
$J_{\mathrm{CC,I}}$; $P_{\mathrm{CC,I}}$	1.40 ; 0.50	Weight modifier for connections from I cue cluster to E opposite cue cluster; Probability of applying modifier

Continuation of Supplementary Table 3		
**Symbol**	**Value**	**Name/Description**
$J_{\mathrm{AC,Corr}}$; $P_{\mathrm{AC,Corr}}$	2.75 ; 0.50	Weight modifier for connections from E cue cluster to appropriate ("correct") E action cluster; Probability of applying modifier
$J_{\mathrm{AC,Inc}}$; $P_{\mathrm{AC,Inc}}$	2.60 ; 0.50	Weight modifier for connections from E cue cluster to inappropriate ("incorrect") E action cluster; Probability of applying modifier
$J_{\mathrm{CA,I}}$; $P_{\mathrm{CA,I}}$	1.40 ; 0.50	Weight modifier for connections from I action clusters to E cue clusters; Probability of applying modifier
$J_{\mathrm{AA,E}}$; $P_{\mathrm{AA,E}}$	1.07 ; 0.50	Weight modifier for connections from E action cluster to itself; Probability of applying modifier
$J_{\mathrm{AA,I}}$; $P_{\mathrm{AA,I}}$	3.00 ; 0.50	Weight modifier for connections from I action cluster to E opposite action cluster; Probability of applying modifier
STIMULUS		
f_{stim}	0.5	Fraction of neurons in taste-selective clusters that respond to external stimulus
G	60%—200%	Maximum stimulus gain (external current modifier)
τ_{R}	150 ms	Stimulus rise time constant
τ_{D}	160 ms—705 ms	Stimulus decay time constant
ACTION GATE		
μ_{ag}	1.5 s	Action gate mean start time
η_{ag}	0.4 s	Halfwidth of uniformly random action gate start time
Δ_{ag}	0.5 s	Time for action gate to increase from 0 to 1
SILENCING PERTURBATION		
κ_{sil}	25%—100%	Silencing strength (external current modifier)
Δ_{sil}	250 ms—2,500 ms	Silencing duration
Δc	50 ms	Increment for sliding center of silencing window

S3 Table. Model parameters. Symbols, values, and brief descriptions for all parameters used in the simulations of the spiking network model presented in the main text.
